# Dyspepsia with Salty Taste

**Published:** 1891-03

**Authors:** J. Kafka

**Affiliations:** Prague


### DYSPEPSIA WITH SALTY TASTE.

Dr. J. Kafka, Prague.

Dr. Moscovitz, of Pesth, in Hungary, enjoying a very large and lucrative practice, and thus constantly overtasking his strength, suffered for a long time from arthritic pains and gastric troubles; his face had a coppery tint, though he never indulged in drinking; nose and cheeks were covered with bluish veins; he felt depressed, his usual good humor had given way to hypochondriasis, and he felt himself very sick. Kafka found spleen and liver considerably swollen, pulsations in epigastrium, bloated abdomen, retarded fæcal discharges, sounds of heart normal, respiration intact, urine acid with frequent sediments. The joints of the shoulders and hands were often very painful, which disturbed his sleep. A season at Carlsbad helped him some, which he had to repeat for several years in order to attend to his professional duties; finally, as his case grew worse again, his friend and counselor was again sent for, as he had lost all appetite on account of a continuous salty taste which disgusted him; he refused nourishment, emaciated, and felt exhausted. Kafka found no gastric symptoms, but only a total loss of appetite on account of the salty taste, while the tongue was clean; no eructations; no oppression or nausea. Considering that, in every arthritic patient, the formation of Sodium salts prevails on the chylics, and when simultaneously Muriatic acid is in too large abundance in the stomach, and both combine to form Natrum-muriaticum, we may find that an explanation for the continuous salty taste of the patient. Following the teachings of Hahnemann and Bœnninghausen, Spiritus nitri dulcis, 1.0 to 100.0 Aque destillata was prescribed, to take a tablespoonful every half hour. He took the first dose at nine A. M., and at noon he was able to enjoy a good lunch, and after a few days he was able to attend again to his practice. Long ago, Kafka read in some journal that in saltworks the laborers often complain of this salty taste, which renders them unable to work, and their physician cures them with Spirits of Nitre.—*Allg. hom. Zeit.*, 23, '90.

Lippe, in his *Repertory*, page 104, gives us for saltish regurgitation: Arn., Sulph-acid, Ant-tart., and for saltish taste: Ars., Brom., *Carb-veg.*, Chin., Cupr., Iod., Lach., Lyc., *Merc.*, Merc-cor., Nitric-acid, Nux-m., Nux-vom., Phos., Puls., Rhus, Sep., Sulph., Therid., *Verat.*, Zinc.

Gentry, in his *Concordance*, II, 155: *Gels.*, dryness in mouth, as if he had eaten salty bacon. *Sepia*, food tastes too salty. *Sulphur*, food tastes too salty, like straw. Everything tastes as if salt: *Carlsbad.* Taste at first mucous, then salty: *Carlsbad.*

We cannot find any proving of sweet Spirits of Nitre in Allen's *Encyclopædia;* he mentions it only not to confound it with Nitric Ether. In fact, in our whole homœopathic literature, we cannot find much on the action or a proving of sweet Spirits of Nitre, and, in the allopathic school, Penzolt and Nothnagel throw it among old lumber and superfluous. Still the older physicians were very fond of it, and their Hoffman's drops were conspicuous in the pharmacies of the grandmothers. The rapid cure which Kafka made after the failure of well-known homœopathic physicians, like Syontagh, brings this drug again to our consideration, and let us hope that other physicians of our school will give us their experience with sweet Spirits of Nitre.

